# Endophytic *Streptomyces* sp. NEAU-ZSY13 from the leaf of *Perilla frutescens*, as a promising broad-spectrum biocontrol agent against soil-borne diseases

**DOI:** 10.3389/fmicb.2023.1243610

**Published:** 2023-08-24

**Authors:** Zhiyan Wang, Congting Gao, Jingquan Yang, Rui Du, Fanli Zeng, Hui Bing, Banghua Xia, Yue Shen, Chongxi Liu

**Affiliations:** ^1^Key Laboratory of Agricultural Microbiology of Heilongjiang Province, Northeast Agricultural University, Harbin, China; ^2^Department of Molecular Pharmacology, Tianjin Medical University Cancer Institute and Hospital, Tianjin, China; ^3^Hebei Technology Innovation Center for Green Management of Soil-borne Diseases, Baoding University, Baoding, China

**Keywords:** soil-borne diseases, endophytic actinobacteria, broad-spectrum antimicrobial activity, biocontrol, niphimycin

## Abstract

Soil-borne diseases cause significant economic losses in global agricultural production. These diseases are challenging to control due to the invasion of multiple pathogens into host plants, and traditional chemical control methods often yield unsatisfactory results. In this study, we isolated and identified an endophytic *Streptomyces*, designated as NEAU-ZSY13, from the leaf of *Perilla frutescens*. This isolate exhibited broad-spectrum antifungal activity against 17 soil-borne phytopathogenic fungi, with *Bipolaris sorokiniana* being the most prominent. Additionally, it displayed strong antibacterial activity against the soil-borne phytopathogenic bacterium *Ralstonia solanacearum*. To assess its biocontrol potential, the isolate was utilized to produce a biofertilizer through solid-state fermentation. The fermentation conditions were optimized using response surface methodology to maximize the spore production. The results revealed that more abundant spores were produced with a 1:2 ratio of vermicompost to wheat bran, 60% water content, 20% inoculation amount and 28°C. Subsequent pot experiments demonstrated that the application of the biofertilizer with a spore concentration of 10^8^ CFU/g soil effectively suppressed the occurrence of tomato bacterial wilt caused by *R. solanacearum* and wheat root rot caused by *B. sorokiniana*, with biocontrol efficacies of 72.2 and 78.3%, respectively. Chemical analysis of NEAU-ZSY13 extracts, using nuclear magnetic resonance spectrometry and mass analysis, identified niphimycin C and niphimycin A as the primary active constituents. These compounds exhibited high activity against *R. solanacearum* (EC_50_ of 3.6 and 2.4 μg mL^−1^) and *B. sorokiniana* (EC_50_ of 3.9 and 3.4 μg mL^−1^). In conclusion, this study demonstrates the potential of *Streptomyces* sp. NEAU-ZSY13 as a biofertilizer for the control of soil-borne diseases.

## Introduction

Soil-borne diseases pose a significant threat to global crop production, resulting in substantial economic losses (Niu et al., 2020). These pathogens can present in the soil or on plant residues for a long time, and infect a wide range of hosts (Fang et al., 2021), and can cause disease complexes that additionally complicate the management of crop diseases (Tzelepis et al., 2021). Simultaneous infection of plants by multiple pathogens, along with their synergistic interactions, can lead to the emergence of new diseases or the exacerbation of symptoms caused by individual pathogens alone (Mangeiro et al., 2022). Consequently, controlling soil-borne diseases affected by multiple pathogens presents a significant challenge.

The extensive use of chemical pesticides in agriculture has raised concerns regarding environmental pollution, pathogen resistance, and potential risks to human and animal health (Mangeiro et al., 2022). As a result, many countries have implemented strict restrictions on the use of chemical pesticides (Kosamu et al., 2020). In China, for instance, the Ministry of Agriculture and Rural Affairs introduced the “Action Plan for Zero Growth of Chemical Fertilizer Use by 2020” in 2015. Since then, pesticide usage has decreased 16.8% by 2021. Biological control is regarded as an effective and environmentally friendly alternative or complement to chemical pesticides for managing fungal and bacterial diseases. Several microbial-based products have been marketed as biocontrol agents, such as *Bacillus subtilis* (QST^®^), *Streptomyces lydicus* WYEC108 (Actinovate^®^), and *Metschnikowia fructicola* (Shemer^®^).

In some cases, plants may be able to recruit beneficial microbes that counteract pathogen assault through a range of mechanisms, including competition for nutrients, production of active compounds, and induction of plant resistance (Liu et al., 2019). Furthermore, the specific ecological niche of endophytes enables them to enhance plant growth and suppress disease more effectively than soil microorganisms. For example, Gao et al. (2021) reported that entophytic actinobacterium *Streptomyces hygroscopicus* OsiSh-2 could promote both yield and disease resistance in host rice. The endophytic bacterium *Pseudomonas* sp. IMBG294 has shown the ability to promote seedling growth and enhance resistance to soft rot in potato (Pavlo et al., 2011). Actinobacteria are well-known for their production of various secondary metabolites, including blasticidin S, kasugamycin, mildiomycin, and validamycin, which have been utilized as agricultural antibiotics to combat plant pathogens (Han et al., 2021). Studies have reported that actinobacteria constitute a dominant group within the plant microbial community (Lundberg et al., 2012). Consequently, endophytic actinobacteria hold great promise as a potential source of biocontrol agents and metabolites to combat soil-borne diseases.

In this study, we isolated an endophytic actinobacterium from *Perilla frutescens*, which exhibited broad-spectrum antimicrobial activity against phytopathogenic fungi and bacteria. We further investigated its potential as a biofertilizer for controlling tomato bacterial wilt caused by *Ralstonia solanacearum* and wheat root rot caused by *Bipolaris sorokiniana*. Additionally, we isolated and identified the active compounds derived from secondary metabolites produced by this microorganism. These findings contribute to the advancement of knowledge regarding the role of endophytic actinobacteria in the management of soil-borne diseases.

## Materials and methods

### Isolation of endophytic actinobacteria from *Perilla frutescens*

The *P. frutescens* samples used in this experiment were collected from Zisu Garden of Huanan Forestry Bureau (Heilongjiang, China). The plant material was divided into root, stem, and leaf sections, and subjected to seven-step surface sterilization procedure as previously described (Liu et al., 2019). After thorough drying under sterile conditions, the samples were ground using a mortar and pestle. Subsequently, 200 μL of the suspension was plated onto five different actinobacteria-selective media types, including Gause’s synthetic (GS) agar no. 1 (Atlas, 1993), sodium succinate-asparagine agar (SSA) (Li et al., 2012), cellulose-proline agar (CPA) (Qin et al., 2009), dulcitol-proline agar (DPA) (Guan et al., 2015), and amino acid agar (AAG) (Liu et al., 2019) and incubated at 28°C for 2–3 weeks. All media were supplemented with nalidixic acid (20 mg/L) and cycloheximide (50 mg/L) to inhibit the growth of Gram-negative bacteria and fungi. Then, the grown colonies were subsequently transferred onto oatmeal agar (ISP3) for further analysis (Shirling and Gottlieb, 1966).

### Screening for antagonistic actinobacteria

Antimicrobial screening was conducted against 17 phytopathogenic fungi and 1 phytopathogenic bacterium, including *Bipolaris sorokiniana* NEAU-W4, *Rhizoctonia solani* CAAS-F11, *Fusarium moniliforme* NEAU-M1, *Fusarium graminearum* PH-1, *Fusarium oxysporum* f. sp. *cucumerinum* CAAS-F13, *Fusarium oxysporum* f. sp. *melonis* CAAS-F14, *Fusarium solani* CAAS-F15, *Fusarium verticillioides* NEAU-C3, *Cochiioboius heterostrophus* CAAS-F27, *Setosphaeria turcica* CAAS-F35, *Corynespora cassiicola* CAAS-F22, *Alternaria tenuissima* NEAU-5, *Alternaria cucumerina* NEAU-7, *Alternaria alternata* NEAU-10, *Verticillium dahlia* CAAS-F43, *Botrytis cinerea* CAAS-F48, *Stagonosporopsis cucurbitacearum* CAAS-F48 and *Ralstonia solanacearum* CAAS-B1. *B. sorokiniana*, *F. moniliforme*, *F. verticillioides*, *A. cucumerina*, *A. tenuissima*, and *A. alternata* were maintained at the Key Laboratory of Agricultural Microbiology of Heilongjiang Province (Harbin, China). *R. solani*, *F. oxysporum* f. sp. *cucumerinum*, *F. oxysporum* f. sp. *melonis*, *F. solani*, *C. heterostrophus*, *S. turcica*, *C. cassiicola*, *S. cucurbitacearum, B. cinerea*, and *R. solanacearum* were obtained from Chinese Academy of Agricultural Sciences (Beijing, China). *F. graminearum* was kindly provided by Zhejiang University (Hangzhou, China). For *R. solanacearum*, the bacterium was cultured on nutrient agar (NA) (Waksman, 1961) at 37°C for 24 h. The antagonistic activity of the isolates against *R. solanacearum* was assessed using the agar cylinders method. A bacterial suspension of 100 μL (OD_600_ = 0.3) was plated onto NA agar. A fresh mycelial agar plug (5 mm diameter) from isolates cultured on ISP3 medium for 7 days was placed in the center of NA plate and incubated at 37°C. The diameter of the inhibition zone was measured after 24 h. Regarding the fungi, they were cultured on potato dextrose agar (PDA) (Cao et al., 2017) at 28°C. The antifungal activity of the isolates were evaluated using the dual culture plate assay (Hamzah et al., 2018). The isolates were point-inoculated at the margin of PDA plates and incubated for 3 days at 28°C, after which a fresh mycelial PDA agar plug of the fungus (5 mm diameter) was transferred to the opposite margin of the corresponding plate. After additional days of incubation at 28°C for 3–7 days, the diameter of the inhibition zones was measured, and the inhibition rates were calculated according to the following formula: (the width of inhibition/the width between pathogen and antagonistic bacteria) × 100%. All experiments were performed in triplicate.

### Phylogenetic analysis of antagonistic actinobacteria

Genomic DNA extraction and PCR amplification of the 16S rRNA gene sequence were performed according to previous study (Wang et al., 2020). The phylogenetic tree was constructed using the neighbor-joining algorithm implemented in Molecular Evolutionary Genetics Analysis (MEGA) software version 11 (Tamura et al., 2021). The stability of the phylogenetic tree topology was assessed using the bootstrap method with 1,000 repetitions (Felsenstein, 1985). An evolutionary distance matrix was calculated according to Kimura’s two-parameter model (Kimura, 1980). All positions containing gaps and missing data were completely eliminated from the dataset. The obtained 16S rRNA gene sequences were aligned using the EzBioCloud server (Yoon et al., 2017).

### Optimization methodology of solid-state fermentation

#### Inoculum preparation

NEAU-ZSY13 was inoculated into 50 mL of GY broth (Jia et al., 2013) and cultured for 3 days at 28°C with agitation at 200 rpm.

#### Substrate ratio optimization

In this experiment, vermicompost and wheat bran were chosen as the base medium in different ratios: 3:1, 2:1, 1:1, 1:2, and 1:3, respectively. The contents were thoroughly mixed and autoclaved at 121°C for 30 min and cooled to room temperature before inoculation. A 10% inoculum was added, and the flasks were incubated at 28°C for 7 days with daily agitation. The spore count was determined using the dilution plate method. The optimal substrate combination was determined based on the highest number of viable colonies observed. The experiments were performed in triplicate.

#### Optimization of inoculum amount, water content, temperature, and pH

A single factor test was employed to assess the effects of different factors on the SSF process of NEAU-ZSY13. The factors investigated included inoculum amount (5, 10, 15, 20, and 25%), water content (40, 50, 60, 70, and 80%), temperature (20, 24, 28, 32, and 34°C), and pH (5, 6, 7, 8, and 9). The experiment was performed in triplicate to ensure reliability and to obtain consistent results.

#### Statistical optimization of biomass production by response surface methodology

To optimize the biomass production of NEAU-ZSY13, we employed a response surface methodology (RSM) based on Box-Behnken design (BBD). Our objective was to optimize the significant factors affecting biomass production, namely moisture content (X_1_), inoculum amount (X_2_), and fermentation temperature (X_3_). The experimental design consisted three factors, each with three repetitions, resulting in a total of 17 primary trials and 51 runs overall. In this design, X_1_, X_2_, and X_3_ were selected as independent variables, while biomass yield (Y) was chosen as the dependent variable. All the statistical analysis, including regression analysis, analysis of variance (ANOVA), contour plots and response surface plots, were carried out using Design Expert 11.6.0 Software (Stat-Ease, Inc., United States).

### Efficacy of NEAU-ZSY13 in controlling tomato bacterial wilt caused by *Ralstonia solanacearum* in growth chamber

The biofertilizer of NEAU-ZSY13 was mixed in the soil (alkaline chernozem) at the final concentrations of 10^6^ CFU/g, 10^7^ CFU/g, and 10^8^ CFU/g of soil. The soil used in the experiment was steam-sterilized at 121°C for 30 min. Tomato seeds (Aoguan 1, moderate susceptible to *R. solanacearum*) were subjected to surface sterilization using 70% ethanol for 1 min, followed by a 2-min soak in 2% NaClO solution. After treatment, the seeds were pregerminated for 2 days at 25°C and then placed in seedling trays under natural conditions. Once they reached the four-leaf stage, the seedlings were transplanted individually into pots containing the biofertilizer, one seedling per pot. After a period of 7 days, a 10 mL suspension of *R. solanacearum* (OD_600_ = 0.3) was applied to the soil surrounding the seedlings. Each treatment consisted of 10 pots, and the entire experiment was repeated three times, independently. These experiments were conducted in a growth chamber set to simulate a 12-h photoperiod. The chamber maintained a temperature of 37°C and humidity level ranging between 75 and 90%. The duration of the experiment was 14 days. The extent of the disease was assessed using a five-class scale, where 0 indicated no wilt, 1 represented 1–25% leaf wilt, 2 denoted 25–50% leaf wilt, 3 indicated 51–75% leaf wilt, and 4 indicated over 75% leaf wilt. To quantify the disease severity, the Disease Index (DI) was computed using the following formula: (Σ(plant numbers with the same rating of disease severity × disease rating)/(maximum rating value × total number of plants)) × 100. Furthermore, the biological control efficiency was calculated using the formula: ((DI of the negative control - DI of the treatment)/DI of the negative control) × 100%.

### Efficacy of NEAU-ZSY13 in controlling wheat root rot caused by *Bipolaris sorokiniana* in growth chamber

The biofertilizer was combined with the alkaline chernozem to achieve spore concentrations of 10^6^ CFU/g, 10^7^ CFU/g, and 10^8^ CFU/g of soil. The soil used in the experiment was steam-sterilized at 121°C for 30 min. The pathogenic fungus *B. sorokiniana* was cultured on wheat grains at 28°C for 10 days to obtain the disease inoculum, then mixed with the soil at a ratio of 1% (w/w). The variety Longmai 33, which is moderately susceptible to *B. sorokiniana*, was selected for the experiment. To prepare the seeds, the seeds were soaked in water for 24 h and subsequently planted in pots containing a mixture of disease inoculum and biofertilizer with different spore concentrations. As a negative control, a treatment without biofertilizer was included. Each treatment consisted of 10 pots, with one seedling per pot. The experiment was replicated three times. The potted plants were maintained at 25°C and a humidity level of 70% for a duration of 14 days. The severity of the disease was evaluated using a five-point scale, corresponding to the percentage of roots and above-ground parts displaying discoloration symptoms: 0 denoted no symptoms, 1 represented 1–25% affected, 2 indicated 26–50% affected, 3 denoted 51–75% affected, and 4 indicated over 75% affected. The DI and biological control efficiency were computed based on the formulas previously mentioned.

### Isolation and characterization of active compounds from NEAU-ZSY13

**Figure 1:**
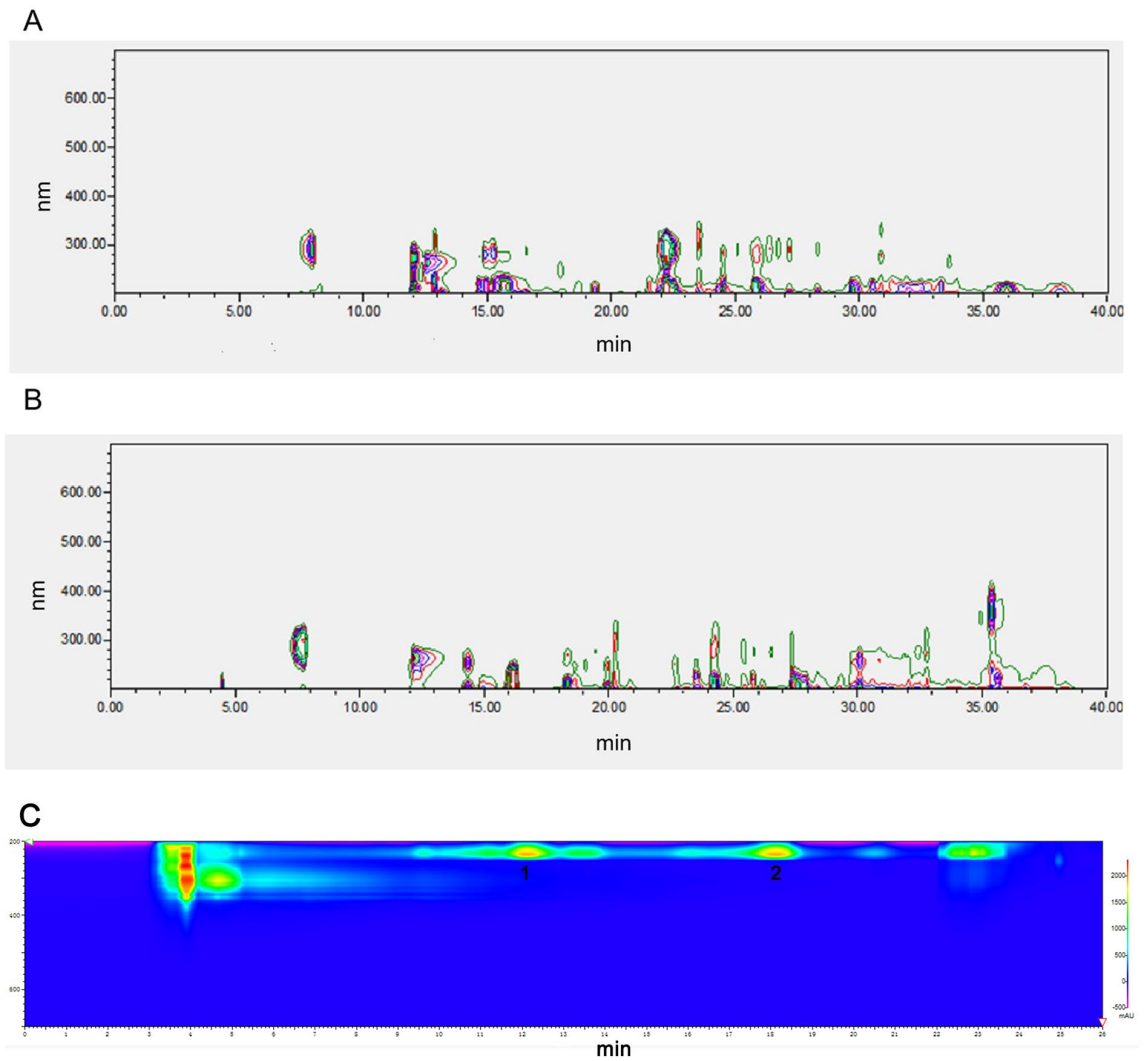
HPLC analysis of fermentation extract from NEAU-ZSY13. **(A)** The extract from supernatant; **(B)** The extract from mycelia: **(C)** Compounds **1** and **2** from F10B.

NEAU-ZSY13 was cultivated on ISP3 medium at a temperature of 28°C for 7 days and then transferred to 250 mL baffled Erlenmeyer flasks containing 50 mL of sterile seed medium (GY). The cultivation was carried out for 3 days at 28°C with shaking at 250 rpm. After that, 18 mL aliquots of the culture were aseptically transferred into 1 L baffled Erlenmeyer flasks containing 300 mL of the production medium. The production medium consisted of malt extract (1%), glucose (0.4%), yeast extract (0.4%), soluble starch (2%), and CaCO_3_ (0.2%) with a pH of 7.2–7.4. The flasks were incubated at 28°C for 1 week with continuous shaking at 250 rpm. Following the fermentation period, the resulting broth (18 L) was centrifuged at 4,000 rpm for 10 min. The supernatant was collected and extracted three times with ethyl acetate. The extract was then evaporated under vacuum, resulting in 8.68 g of crude oily extract. The mycelia were extracted with methanol (1 L), and the resulting extract was concentrated under vacuum to remove the methanol. This process led to the production of 25.13 g of concentrated extract. Both of these extracts exhibited identical sets of metabolites, as confirmed by High-Performance Liquid Chromatography (HPLC) analysis (Figures 1A,B). Due to the similarity in metabolite profiles, the two extracts were combined for subsequent purification steps. The crude extract was subjected to column chromatography using a silica gel column (100–200 mesh). Elution was performed using a series of solvents, starting with petroleum ether/ethyl acetate mixtures (5/1, 1/1, 1/5, 1/10, and 0/1, v/v), followed by ethyl acetate/methanol mixtures (10/1, 5/1, 1/1, 1/5, and 1/10, v/v). This process resulted in the separation of 10 fractions labeled as fractions 1 through 10. Notably, fraction 10 demonstrated bioactivity. Subsequently, fraction 10 was further purified using a Sephadex LH-20 column, eluting with methanol. This step resulted in the collection of three sub-fractions denoted as F10A, F10B, and F10C. Among these, the fraction displaying bioactivity was F10B. Subsequently, F10B was subjected to semipreparative HPLC using a YMC-Triart C18 column. The mobile phase was a mixture of methanol and water (78/22 v/v) at a flow rate of 3 mL/min. This yielded compounds **1** (retention time, *t*_R_ = 11.5 min, 8 mg) and **2** (*t*_R_ = 17.5 min, 8.8 mg) (Figure 1C). The structures of compounds **1** and **2** were determined through spectroscopic analysis. NMR spectra were acquired using a Bruker Avance III-400 spectrometer in CD_3_OD, with TMS as the internal standard. ESI-MS data were obtained using an Agilent G6230 Q-TOF mass spectrometer.

### Activity evaluation of compounds 1 and 2 against *Ralstonia solanacearum* and *Bipolaris sorokiniana in vitro*

To assess the antibacterial activity of compounds **1** and **2** against *R. solanacearum*, a bacterial suspension (30 μL) was inoculated into 5 mL of NA broth containing different concentrations of the compounds. The tubes were then incubated at 37°C for 24 h, and the OD_600_ was measured. The tube containing the same amount methanol was used as a negative control. The assay was conducted in triplicate to ensure accuracy and reliability of the results. The percentage of inhibition was calculated using the formula [1 - (mean OD_600_ value of the treatment/mean OD_600_ value of the negative control)] × 100%. Linear regression analysis was conducted on the data to determine the effective concentrations required for 50% inhibition (EC_50_).

For the antifungal activity assay against *B. sorokiniana*, compounds **1** and **2** were dissolved in methanol and then diluted to various concentrations with water. These solutions were added to PDA medium. A fungal plug measuring 5 mm in diameter, taken from *B. sorokiniana*, was placed at the center of each PDA plate, which was then incubated at 28°C for 7 days. A control plate containing the same amount methanol was used as a negative control. The experiments were performed in triplicate to validate the findings. The percentage of inhibition was calculated using the formula [1 - (mean colony diameter of the treatment/mean colony diameter of the negative control)] × 100%. Linear regression analysis was conducted on the data to determine the EC_50_ value.

### Statistical analysis

All experiments were conducted in triplicate, and all data were presented as the mean values ± standard deviation. Data processing and statistical analysis were performed using SPSS statistical software package (SPSS Inc., Cary, NC, United States, v.26). Multiple comparisons were performed on the data using a one-way ANOVA program, followed by a Waller-Duncan multiple range test. Differences were considered significant when the probability was less than 0.05.

## Results

### Isolation and identification of NEAU-ZSY13 with broad-spectrum antimicrobial activity

**Figure 2:**
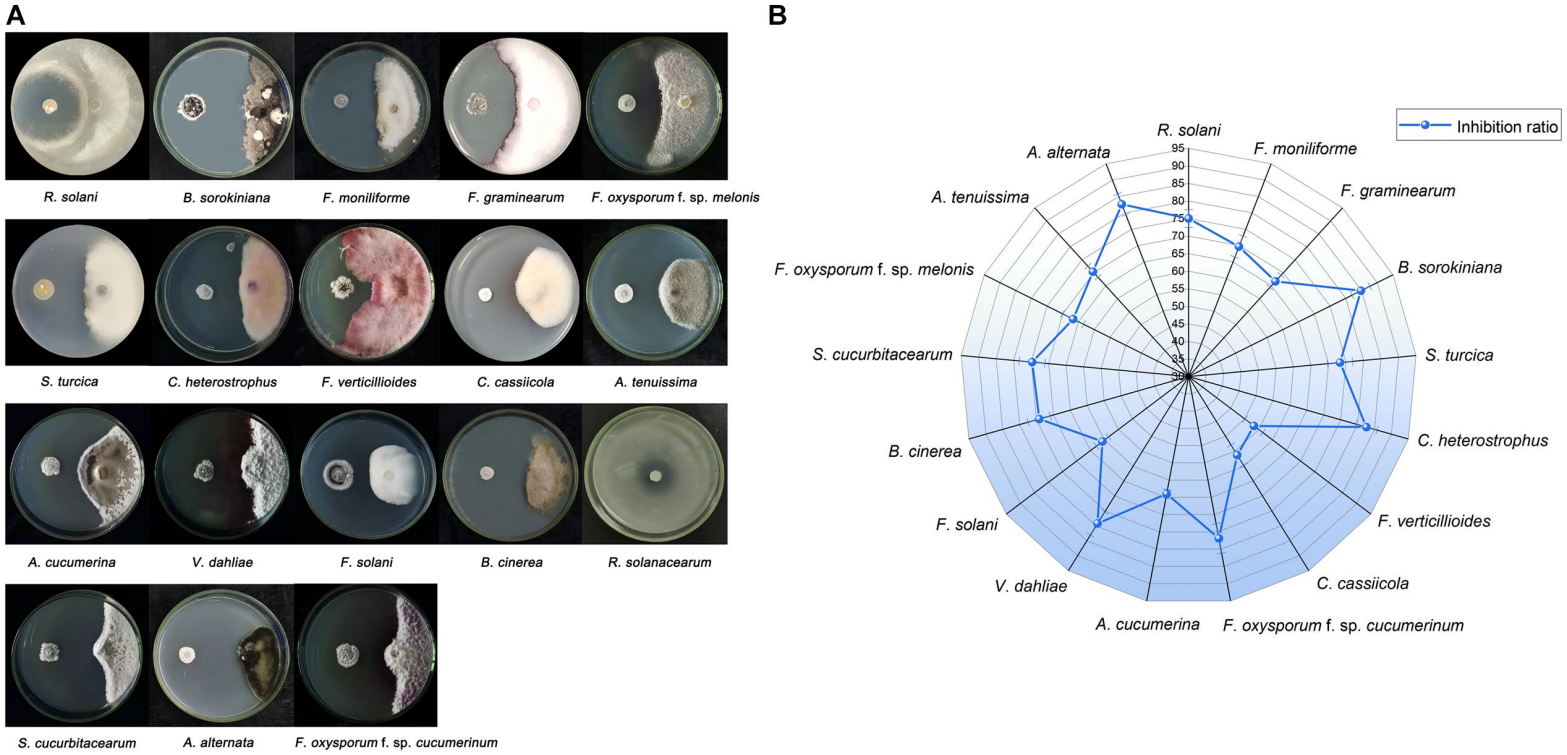
The antimicrobial activity of NEAU-ZSY13 against soil-borne pathogens. **(A)** The antagonistic activity of NEAU-ZSY13 against 17 fungi and *R. solanacearum.* **(B)** The inhibition rate of NEAU-ZSY13 against 17 fungi.

A total of 76 isolates were obtained from *P. frutescens*, comprising 24 from leaf, 17 from stem, and 35 from root. Among these, one particular isolate, NEAU-ZSY13, retrieved from leaf using GS no. 1 medium, displayed robust antagonistic activity against all tested fungi, showcasing inhibition rates spanning 53.3–84.8% (Figure 2). Notably, it exhibited remarkable antifungal potency against *B. sorokiniana*. Additionally, NEAU-ZSY13 suggested potent antibacterial capability against *R. solanacearum*, marked by a 24.3 mm inhibition diameter (Figure 2A).

**Figure 3:**
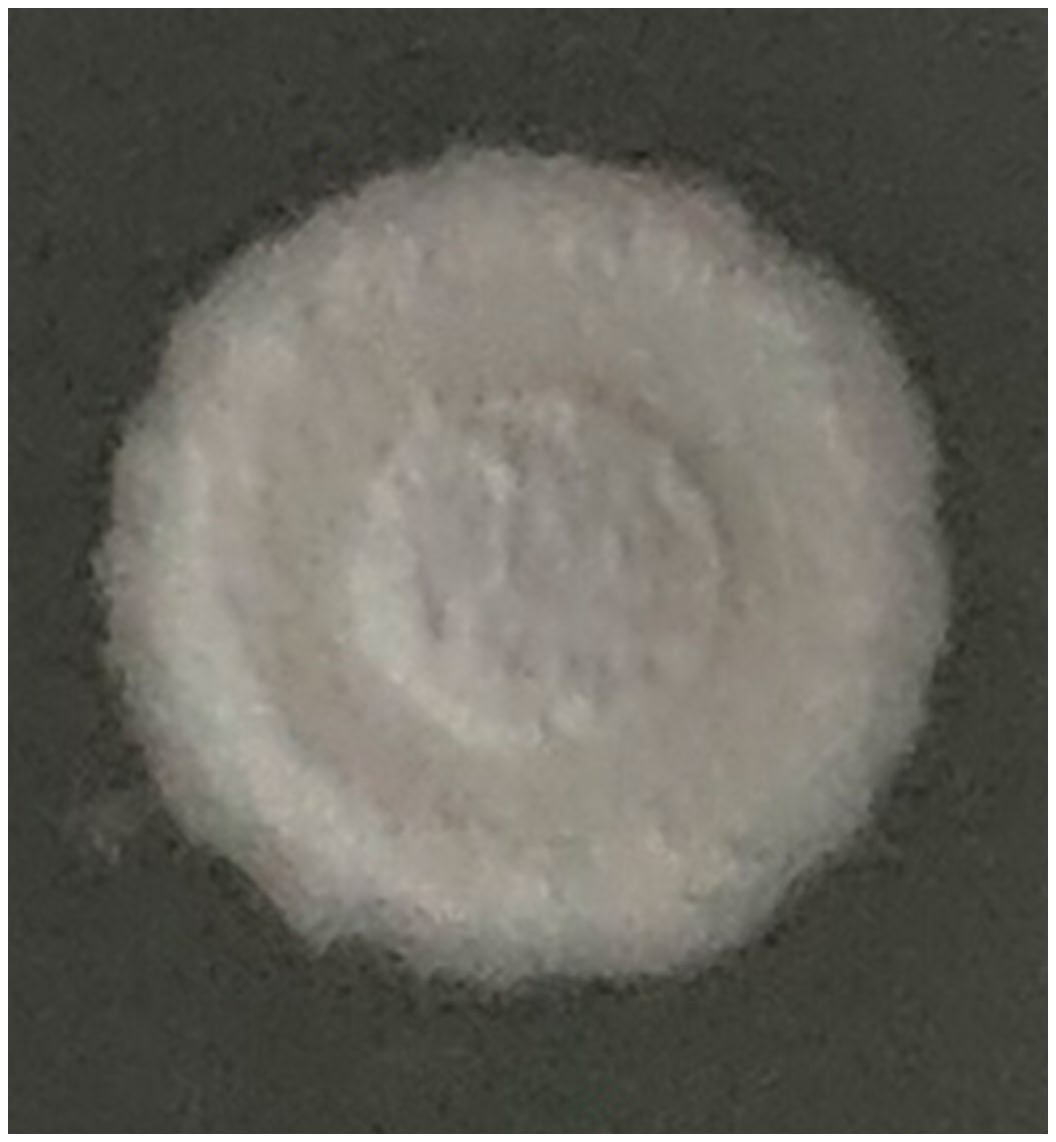
Colony morphology of NEAU-ZSY13 grown on ISP3 medium for 1 week.

**Figure 4:**
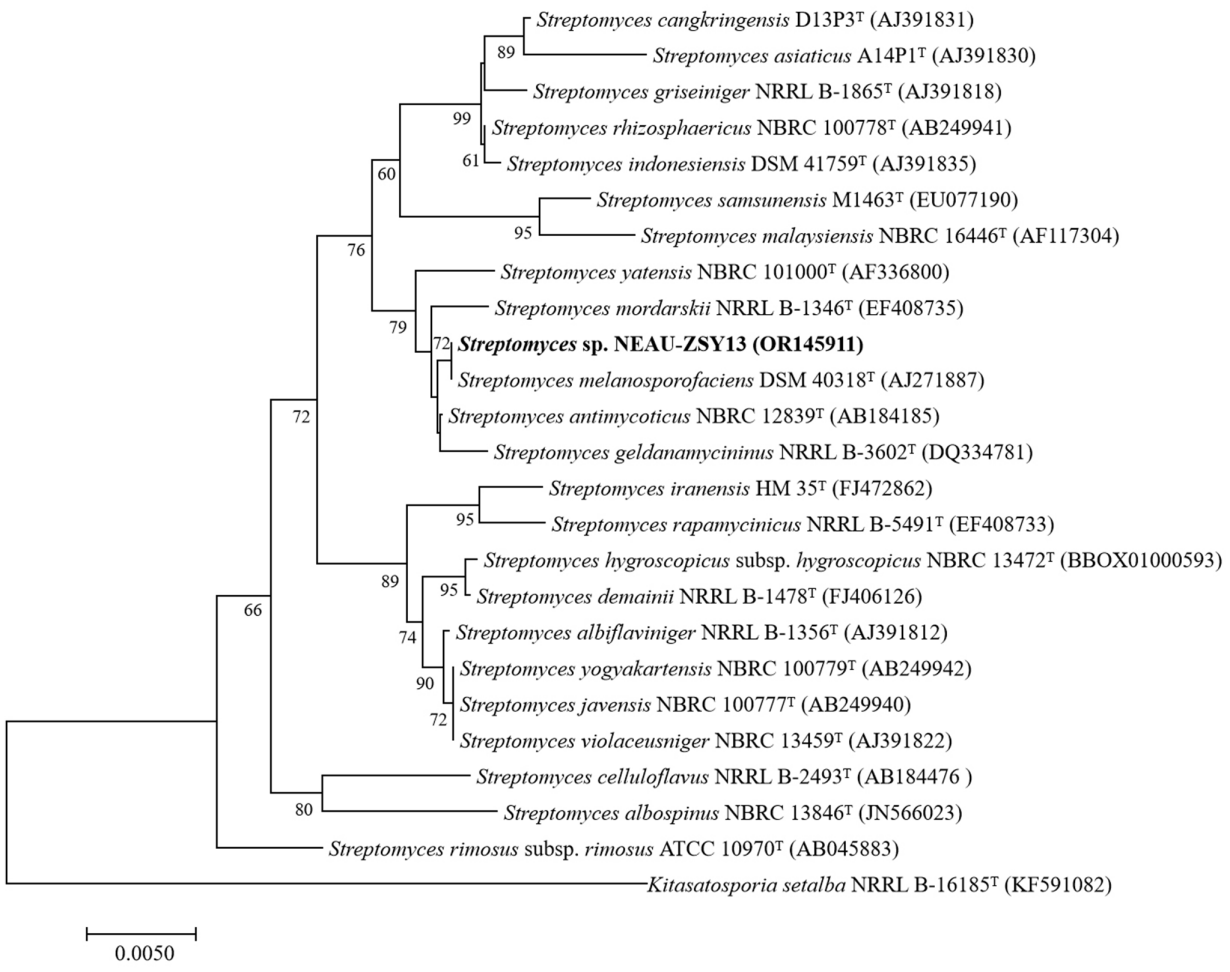
Neighbor-joining tree based on 16S rRNA gene sequences showing relationships between NEAU-ZSY13 and phylogenetically closely related representative species with validly-published names in the genus *Streptomyces*. Numbers at the nodes are percentage bootstrap values based on a neighbor-joining analysis of 1,000 sampled datasets, only values above 50% are given. The out-group used was *Kitasatospora setae* NRRL B-16185^T^; Bar, 0.005 nucleotide substitutions per site.

The morphology of NEAU-ZSY13 exhibited typical characteristics of the genus *Streptomyces*, with white-colored mycelia when cultured on ISP3 medium at 28°C for 7 days (Figure 3). Elucidation of the 16S rRNA gene sequence provided unequivocal substantiation of its taxonomic classification within the genus *Streptomyces*, revealing a maximal sequence identity of 100% with *Streptomyces melanosporofaciens* DSM 40318. In the 16S rRNA-based phylogenetic tree (Figure 4), NEAU-ZSY13 formed a subclade with *S. melanosporofaciens* DSM 40318. Hence, drawing from these findings, NEAU-ZSY13 has been classified as a species within the genus *Streptomyces*.

### Biofertilizer preparation by SSF

In this study, the growth of NEAU-ZSY13 was investigated using wheat bran and vermicompost as solid fermentation substrates. Through systematic analysis, the optimal substrate ratio was identified as a 1:2 (vermicompost to wheat bran by mass), yielding a spore production of 2.27 × 10^9^ CFU/g (Supplementary Figure S1A).

Results from the single-factor tests revealed significant influences of water content, inoculum amount, and temperature on spore production, with pH exerting minor effect (Supplementary Figures S1B–E). Based on these findings, water content, inoculum amount, and temperature were selected as fixed variables for the subsequent BBD. The BBD encompassed 17 runs, systematically investigating the impact of each factor on spore production (Supplementary Table S1). The biomass yield was measured as the response variable, and the obtained data were fitted to a polynomial equation as follows:

$\begin{array}{l} Y=9.49+0.205\;X_{1}+0.1163\;X_{2}+0.0662\;X_{3}-\\ 0.0025\;X_{1}X_{2}+0.0575\;X_{1}X_{3}+\\ 0.0050\;X_{2}X_{3}-0.9875\;X_{1}{\;}^{2}-\\ 0.3500\;X_{2}{\;}^{2}-0.4200\;X_{3}{\;}^{2} \end{array}$

**Figure 5:**
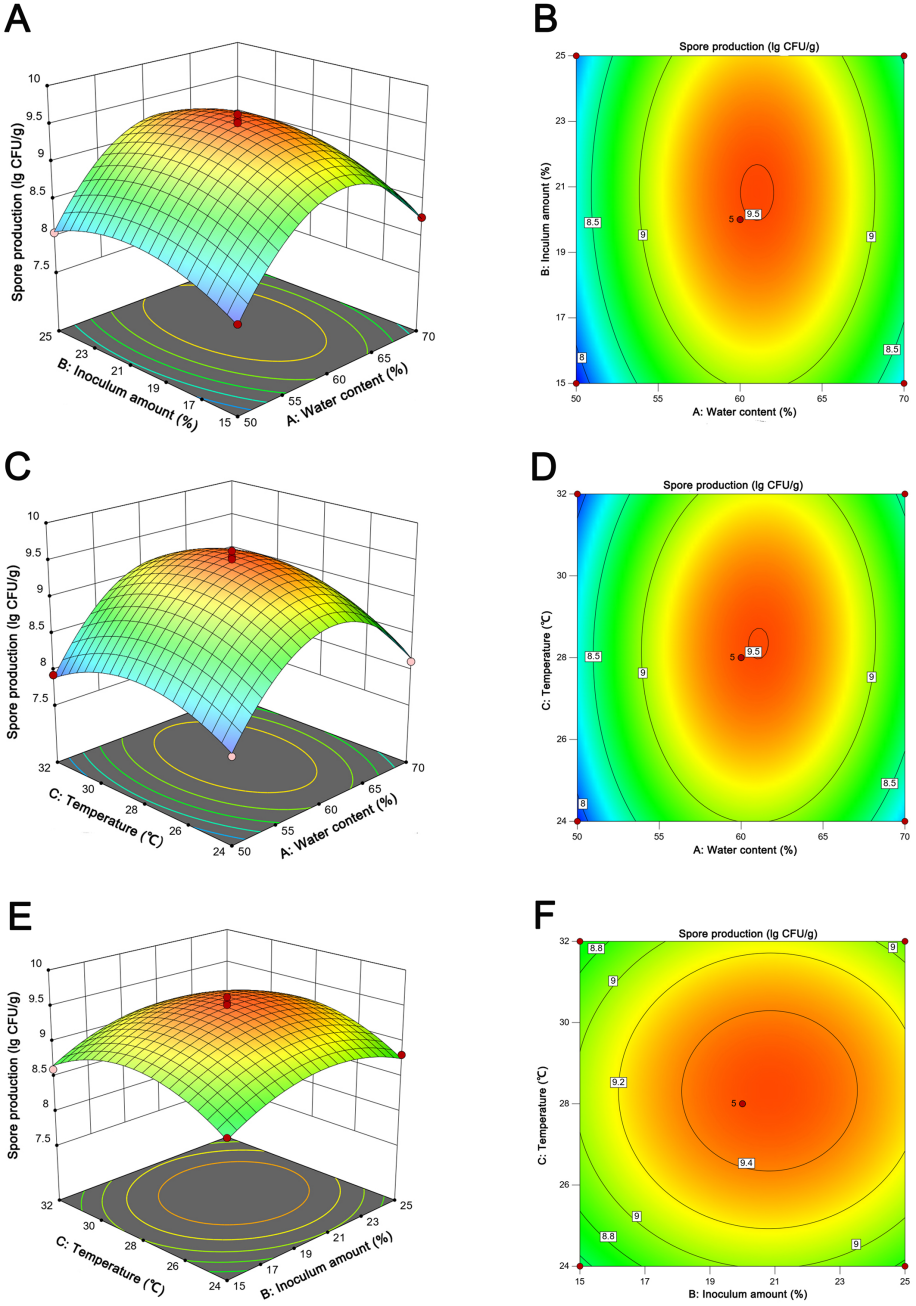
Response surface of spore production from NEAU-ZSY13 showing the interactive effects of the inoculation amount vs. water content **(A,B)**; temperature vs. water content **(C,D)**; temperature vs. temperature **(E,F)**.

The statistical significance of the experimental model was rigorously evaluated through the application of ANOVA, as elucidated in Supplementary Table S2. Factors with a *p*-values beneath the 0.05 threshold were designated as statistically significant. Evidently, the model exhibited a pronounced degree of congruence, as evidenced by the coefficient of determination (*R*^2^) value of 0.9888. This value substantiates a robust correlation between the empirical and predicted outcomes. Furthermore, the lack of fit test rendered an insignificantly elevated *p-*value of 0.7878 (>0.05), thereby attesting to the model’s commendable accuracy. Elucidating the intricate interrelationships among variables, the 3D response surface plots and contour lines presented in Figure 5 provided a graphical medium for the visualization of the optimal fermentation conditions. Through a meticulous statistical analysis, the anticipated optimal parameters for fermentation were pinpointed: 61.1% water content, 20.8% inoculum amount, and 28.3°C. This specific combination was projected to yield the zenith of spore production, quantified at 3.18 × 10^9^ CFU/g. Subsequently, the projected parameters derived from the application of the RSM were subjected to empirical validation. To align with practical operational considerations, the fermentation conditions were judiciously calibrated to encompass 60% water content, 20% inoculum amount, and 28°C. The ensuing spore production quantified under these optimized parameters amounted to 3.29 ± 0.41 × 10^9^ CFU/g, demonstrating a close concurrence with the forecasted value. Furthermore, an optimal fermentation period of 7 days was established (Supplementary Figure S1F).

### Effect of NEAU-ZSY13 on controlling tomato bacterial wilt caused by *Ralstonia solanacearum*

**Figure 6:**
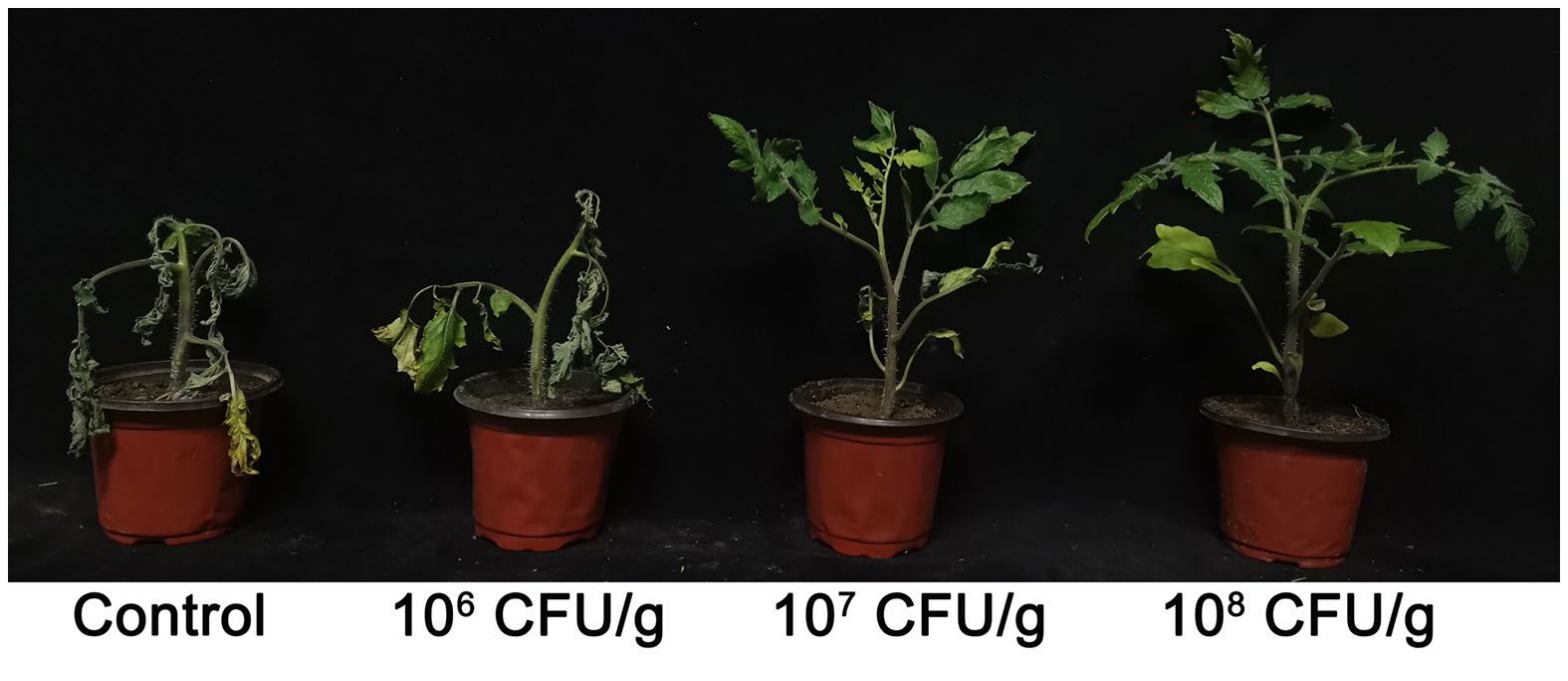
Effect of biofertilizer with NEAU-ZSY13 on growth of tomato plants infected with *R. solanacearum.*

The application of the NEAU-ZSY13-based biofertilizer yielded a significant reduction in the severity of tomato bacterial wilt as compared to the negative control. Notably, the degree of disease severity exhibited a proportional decrease with the increasing spore concentration of the biofertilizer in the soil. Further elucidation of this relationship can be found in Table 1; Figure 6. Specifically, the achievement of a spore concentration of 10^8^ CFU/g within the soil corresponded to a noteworthy biocontrol efficacy of 72.15%. This compelling observation underscores the substantial potential of the biofertilizer at this particular concentration to effectively manage the growth and dissemination of tomato bacterial wilt.

**Table 1:** Effects of biofertilizer with NEAU-ZSY13 on disease protection of tomato seedlings inoculated with *R. solanacearum*.

Treatments	Disease incidence (%)	Control efficacy (%)
Control	93.28 ± 2.17a	/
10^6^ CFU/g	72.70 ± 3.29b	22.06 ± 1.19c
10^7^ CFU/g	35.20 ± 2.98c	62.26 ± 1.17b
10^8^ CFU/g	25.98 ± 1.76d	72.15 ± 2.1a

Data with different lowercase letters are significantly different at the 0.05 probability level.

### Effect of NEAU-ZSY13 on controlling wheat root rot caused by *Bipolaris sorokiniana*

**Figure 7:**
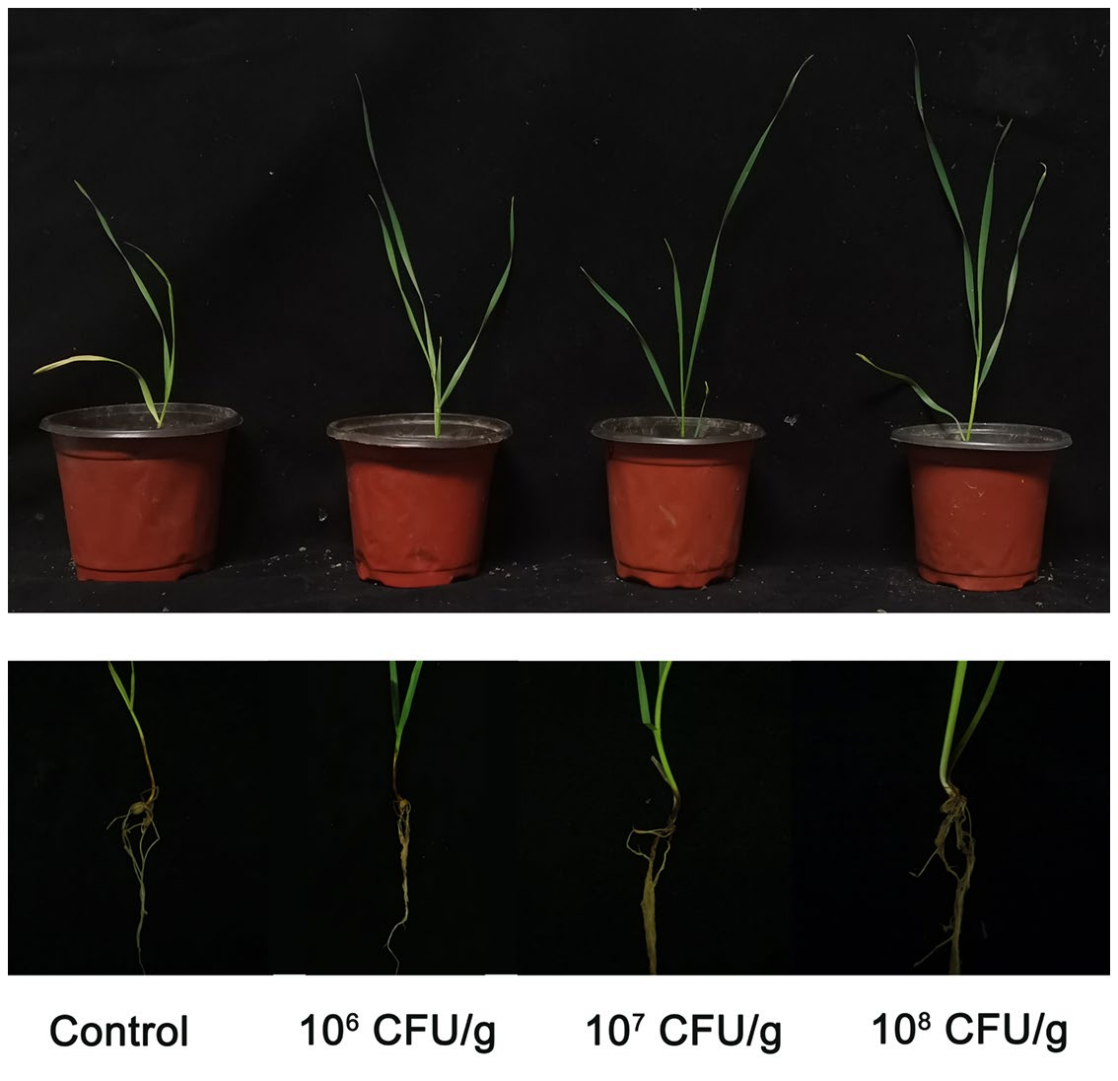
Effect of biofertilizer with NEAU-ZSY13 on growth of wheat plants infected with *B. sorokiniana.*

After a growth period of 14 days within the experimental pots, we conducted a comprehensive evaluation of wheat root rot incidence and concurrently assessed the efficacy of the implemented biological control interventions. As visually depicted in Figure 7, the cohort designated as the negative control, comprising wheat seedlings deliberately inoculated with *B. sorokiniana*, exhibited distinct and conspicuous black necrotic lesions both on the subterranean root system and the above-ground portions, leading to manifest limitations in growth and development. Conversely, the application of NEAU-ZSY13 exhibited a notable mitigation of disease severity, as discerned from the visibly reduced necrotic manifestations. The investigation further revealed that the employment of a spore concentration of 10^8^ CFU/g within the soil resulted in the attainment of the highest level of biocontrol efficacy, reaching an impressive value of 78.28% (Table 2). These empirical findings underscore the substantial potential of NEAU-ZSY13 as an efficacious biological control agent for curtailing wheat root rot, substantiating its prospective utility in managing the disease in agrarian contexts.

**Table 2:** Effects of biofertilizer with NEAU-ZSY13 on disease protection of wheat seedlings inoculated with *B. sorokiniana*.

Treatments	Disease incidence (%)	Control efficacy (%)
Control	79.20 ± 2.78a	/
10^6^ CFU/g	39.40 ± 2.54b	50.25 ± 1.39c
10^7^ CFU/g	31.41 ± 3.24c	60.34 ± 1.6b
10^8^ CFU/g	17.20 ± 3.28d	78.28 ± 0.91a

Data with different lowercase letters are significantly different at the 0.05 probability level.

### Identification and activity evaluation of the active compounds

**Figure 8:**
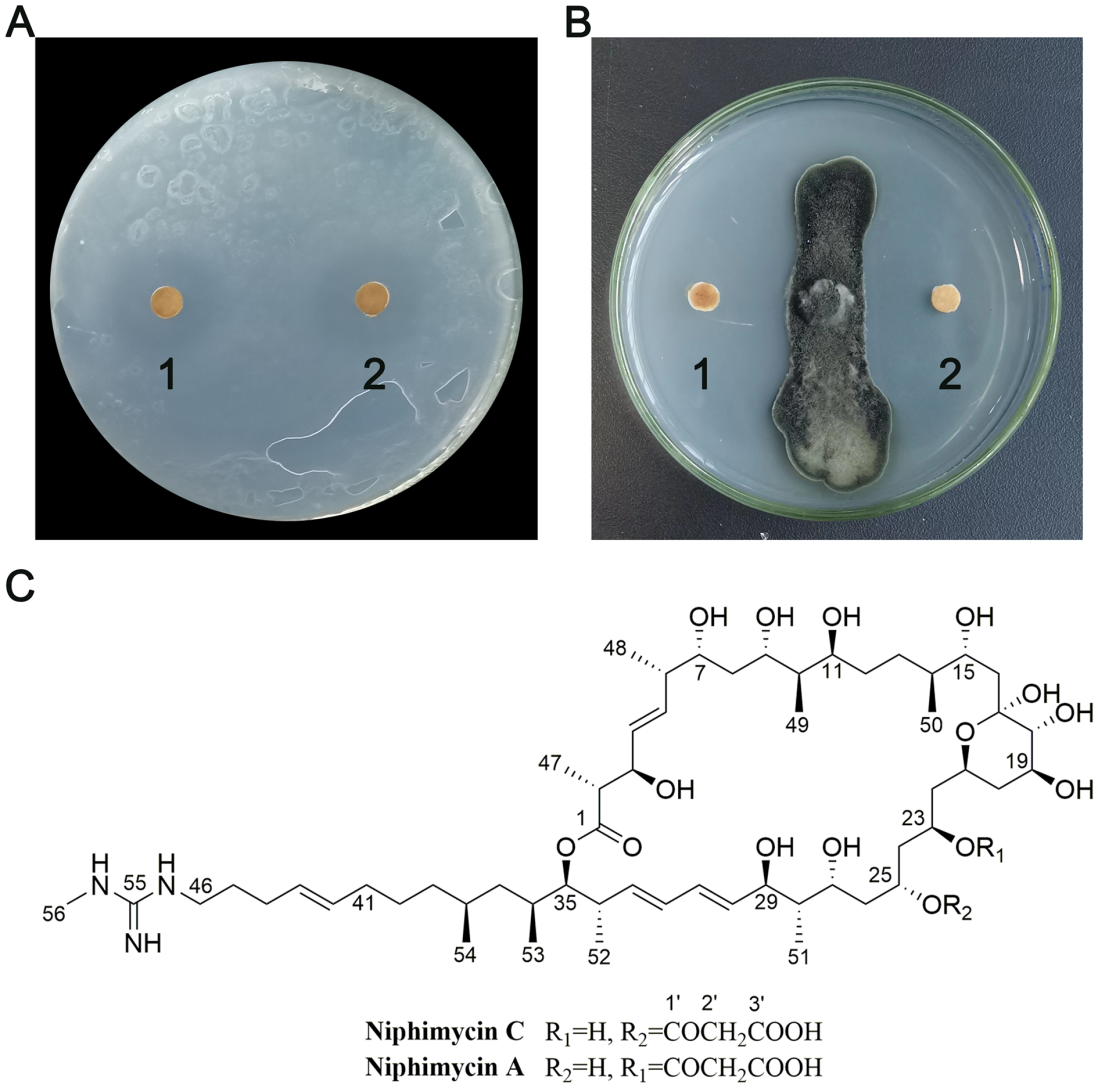
Antimicrobial activity and structures of compounds **1** and **2. (A)** Antibacterial activity of compounds **1** and **2** against *R. solanacearum*; **(B)** Antifungal activity of compounds **1** and **2** against *B. sorokiniana*; **(C)** The structures of compounds **1** and **2**.

This study employed an activity-guided approach to isolate biologically active compounds from NEAU-ZSY13, leading to the identification of two distinct compounds (Figure 8). The determination of EC_50_ values revealed that compounds **1** and **2** exhibited significant activity against *R. solanacearum*, with values of 3.9 and 3.4 μg/mL, and against *B. sorokiniana*, with values of 3.6 and 2.4 μg/mL, respectively.

Compound **1** was obtained as a white crystal. Its ESIMS spectrum showed a molecular ion peak at *m/z* 1,142 [M + H]^+^ (Supplementary Figure S2). ^1^H NMR (600 MHz, CD_3_OD) and ^13^C NMR (150 MHz, CD_3_OD) NMR data were shown in Supplementary Table S3; Supplementary Figures S3, S4. Through meticulous comparison with previously study (Hu et al., 2018), the identity of compound **1** was confirmed as niphimycin C.

Compound **2** was obtained as a white crystal. Its ESIMS spectrum showed a molecular ion peak at *m/z* 1,142 [M + H]^+^ (Supplementary Figure S5). ^1^H NMR (600 MHz, CD_3_OD) and ^13^C NMR (150 MHz, CD_3_OD) NMR data were shown in Supplementary Table S3; Supplementary Figures S6, S7, substantiated its characterization as niphimycin A (Hu et al., 2018).

## Discussion

Soil-borne diseases present a formidable challenge due to the intricate nature and persistence of the causative pathogens (Tollenaere et al., 2016). The utilization of microorganisms for the management of soil-borne diseases offers a multifaceted approach that not only effectively addresses the diseases at their origin but also aligns with the principles of microecology, ensuring safety and reliability. This strategy boasts several advantages, including economical control, environmental compatibility, and the absence of pesticide residues. Consequently, it has emerged as a focal point in soil-borne disease prevention and management research (Poveda et al., 2020). Numerous investigations have demonstrated that the application of microorganisms to plants can significantly retard the onset of soil-borne disease symptoms and mitigate disease severity (Zhuang et al., 2020; Jia et al., 2021). In this particular study, an endophytic *Streptomyces* designated as NEAU-ZSY13 was isolated from the leaf of *P. frutescens*. Remarkably, this strain exhibited an expansive spectrum of antifungal activity, effectively targeting 10 genera of phytopathogenic fungi capable of affecting various crops such as cucumber, tomato, melon, rice, wheat, and corn. Moreover, it demonstrated potent antibacterial activity against the phytopathogenic bacterium *R. solanacearum*. This comprehensive antimicrobial profile positions the isolated *Streptomyces* strain as a promising candidate for exploitation as a biocontrol agent in the management of soil-borne diseases.

Microbial biofertilizers have garnered global attention and acceptance due to their pivotal role in enhancing agricultural productivity and product quality. Numerous microorganisms have found application as biofertilizers, encompassing nitrogen-fixing soil bacteria (*Rhizobium* and *Azotobacter*), arbuscular mycorrhizal (AM) fungi, and antagonistic bacteria (*Streptomyces* and *Bacillus*) (Olanrewaju and Babalola, 2019; Sumbul et al., 2020; Luo et al., 2022). Among these, *Streptomyces* species have gained prominence as biocontrol agents, offering a direct application to crops in the form of culture filtrate or spore suspension (Zhuang et al., 2020; Zhang et al., 2021). Nonetheless, the practical utilization of culture filtrates or spore suspensions is accompanied by challenges related to stability during storage and logistical constraints in large-scale deployment. To address these concerns, the present study harnessed the potential of NEAU-ZSY13 by formulating it into a microbial biofertilizer. This innovative approach was undertaken to evaluate the capacity of NEAU-ZSY13 for controlling soil-borne diseases. Such a formulation not only provides stability advantages over culture filtrates or spore suspensions but also aligns with the exigencies of large-scale applications, offering a promising avenue for bolstering soil-borne disease management strategies.

The choice of raw materials employed as substrates holds pivotal significance in furnishing nutrients for microbial cultivation and the production of secondary metabolites within SSM systems. Vermicompost, an organic material enriched with nutrients, is the outcome of earthworm-mediated decomposition in mesothermal processes and stands as an optimal substrate for the SSF of *Streptomyces* (Shen et al., 2016). This study’s outcomes align with our findings, showcasing that growth media supplemented with vermicompost demonstrated pronounced spore production. The influence of temperature and water content during SSF on growth and spore formation cannot be understated, with optimal values significantly boosting spore yield. This observation corroborates with earlier research indicating that the ideal temperature for SSF profoundly impacts the growth of antagonistic *Streptomyces hygroscopicus* B04 (Shen et al., 2016).

B. *sorokiniana* ranks among the most destructive pathogens affecting wheat, instigating common root rot across diverse wheat varieties (Zhang et al., 2020). Similarly, *R. solanacearum*, a soil-residing phytopathogen accountable for bacterial wilt disease across over 200 plant species, stands as a highly detrimental phytopathogenic bacterium (Mansfield et al., 2012). With these considerations, our study opted for *B. sorokiniana* and *R. solanacearum* as representative targets of fungi and bacteria, respectively, aiming to comprehensively assess the disease control efficacy of the formulated biofertilizer. The outcomes revealed a noteworthy trend: augmented spore concentrations corresponded to heightened disease control effectiveness exhibited by the biofertilizer against these distinct pathogens. This observation substantiates the pivotal contribution of NEAU-ZSY13 within the biofertilizer matrix, affirming its central role in the biofertilizer’s disease-controlling potential. Consequently, the demonstrated efficacy presents a promising application trajectory for the biofertilizer in soil-borne disease management strategies.

A plethora of *Streptomyces* species have been documented for their proficiency in producing diverse antibiotics, thereby exhibiting robust antimicrobial efficacy against a wide spectrum of plant pathogens (Alam et al., 2022). In pursuit of comprehending the antimicrobial mechanism exhibited by NEAU-ZSY13, the isolation of two bioactive compounds, namely niphimycin C and niphimycin A, was undertaken. Niphimycin A, alternately recognized as niphimycin Iα or scopafungin, has garnered attention for its wide-ranging antibacterial and antifungal attributes. Among its achievements, it has demonstrated activity against several phytopathogenic fungi, encompassing *Alternaria mali*, *Aspergillus oryzae*, *Botrytis cinerea*, *Cercospora canescens*, *Colletotrichum coccodes*, *Colletotrichum gloeosporioides*, *Colletotrichum orbiculare*, *Cylindrocarpon destructans*, *Fusarium oxysporum* f. sp. *cucumerinum*, *Fusarium oxysporum* f. sp. *lycopersici*, and *Rhizoctonia solani* (Samain et al., 1982; Laskowski et al., 2012). Recently, niphimycin A, along with niphimycin C, was rediscovered from a marine-derived *Streptomyces*, and their absolute configurations were completely resolved (Hu et al., 2018). Their pronounced antibacterial activity was exemplified against methicillin-resistant *Staphylococcus aureus*, showcasing a minimum inhibitory concentration (MIC) of 8 μg/mL, which attests to their potent antimicrobial potential.

## Conclusion

In summary, the isolated *Streptomyces* sp. NEAU-ZSY13, originating from the leaf of *P. frutescens*, has showcased remarkable antimicrobial prowess against soil-borne pathogens. The application of a biofertilizer derived from this strain, as evidenced by pot experiments, displayed substantial effectiveness in mitigating the incidence of tomato bacterial wilt, triggered by *R. solanacearum*, and wheat root rot, caused by *B. sorokiniana*. Furthermore, the isolation of bioactive compounds, identified as niphimycin C and niphimycin A, from the secondary metabolites of this strain adds to its antimicrobial repertoire. Moving forward, our research trajectory involves undertaking safety assessments and field experiments to comprehensively gage the influence of this microbial biofertilizer on soil-borne disease management strategies.

## Data availability statement

The original contributions presented in the study are included in the article/Supplementary material, further inquiries can be directed to the corresponding authors.

## Author contributions

CL and YS designed the experiments. ZW, CG, JY, RD, HB, FZ, and BX performed the experiments. CL, ZW, and CG analyzed the data and prepared the manuscript. YS reviewed the manuscript. All authors contributed to the article and approved the submitted version.

## Funding

This work was supported by Hebei Technology Innovation Center for Green Management of Soil-borne Diseases (Baoding University) (2022K02) and Postdoctoral Start-up Fund of Heilongjiang Province (LBH-Q19011 and LBH-Q19082).

## Conflict of interest

The authors declare that the research was conducted in the absence of any commercial or financial relationships that could be construed as a potential conflict of interest.

## Publisher’s note

All claims expressed in this article are solely those of the authors and do not necessarily represent those of their affiliated organizations, or those of the publisher, the editors and the reviewers. Any product that may be evaluated in this article, or claim that may be made by its manufacturer, is not guaranteed or endorsed by the publisher.
