# Supplementary material for: Endophytic Streptomyces sp. NEAU-ZSY13 from the leaf of Perilla frutescens, as a promising broad-spectrum biocontrol agent against soil-borne diseases
Source: Front Microbiol. 2023 Aug 24;14:1243610. doi: 10.3389/fmicb.2023.1243610 (PMC10483227; doi:10.3389/fmicb.2023.1243610)
Supplement: Supplementary file 1 [file Data_Sheet_1.PDF]

Supporting information

Endophytic *Streptomyces* sp. NEAU-ZSY13 from the Leaf of *Perilla frutescens*, as A Promising Broad-Spectrum Biocontrol Agent Against Soil-borne Diseases

Zhiyan Wang[1,2†], Congting Gao[1,3,†], Jingquan Yang[1,†], Rui Du[1], Fanli Zeng[1], Hui Bing[1], Banghua Xia[1], Yue Shen[1*], Chongxi Liu[1,3*]

[1]Key Laboratory of Agricultural Microbiology of Heilongjiang Province, Northeast Agricultural University, Harbin 150030, China

[2]Department of Molecular Pharmacology, Tianjin Medical University Cancer Institute & Hospital, Tianjin 300072, China

[3]Hebei Technology Innovation Center for Green Management of Soil-borne Diseases, Baoding University, Baoding 071000, China

Corresponding authors

E-mail addresses: shenyue@neau.edu.cn (Y. Shen); liuchongxi@neau.edu.cn (C. Liu).

[†]These authors contributed equally to this work.

Running title: "Endophytic *Streptomyces* Control Soil-Borne Diseases"

Table S1. The BBD matrix and experimental data for the response.

Run	Factor 1: X_1: Water content (%)	Factor 2: X_2: Inoculum amount (%)	Factor 3: X_3: Temperature (℃)	Response: Y: Spore production lg CFU/g
1	50	15	28	7.84
2	70	15	28	8.26
3	50	25	28	8.05
4	70	25	28	8.46
5	50	20	24	7.83
6	70	20	24	8.12
7	50	20	32	7.93
8	70	20	32	8.45
9	60	15	24	8.57
10	60	25	24	8.82
11	60	15	32	8.61
12	60	25	32	8.88
13	60	20	28	9.43
14	60	20	28	9.55
15	60	20	28	9.63
16	60	20	28	9.52
17	60	20	28	9.32

Table S2. Analysis of variance (ANOVA) for the Quadratic model.

Response: spore production					
Source	Sum of Squares	df	Mean Square	F-value	p-value
Model	6.23	9	0.7018	68.44	<0.0001**
X_1-Water content	0.3362	1	0.3362	32.79	0.0007
X_2-Inoculum amount	0.1081	1	0.1081	10.54	0.0141
X_3-Temperature	0.0351	1	0.0351	3.42	0.1067
X_1X_2	0.0000	1	0.0000	0.0024	0.9620
X_1X_3	0.0132	1	0.0132	1.29	0.2935
X_2X_3	0.0001	1	0.0001	0.0098	0.9241
X_1^2	4.11	1	4.11	400.44	<0.0001**
X_2^2	0.5158	1	0.5158	50.30	0.0002**
X_3^2	0.7427	1	0.7427	72.44	<0.0001**
Residual	0.0718	7	0.0103		
Lack of Fit	0.0152	3	0.0051	0.3575	0.7878
Pure Error	0.0566	4	0.0142		
Cor Total	6.39	16			
R^2	0.9888				
Adj R^2	0.9743				
Pred R^2	0.9481				

Table S3. ^{1}H NMR and ^{13}C NMR data of compounds **1** and **2** in CD_3OD.

No	**1**		**2**	
	δ_C	δ_H (*J* in Hz)	δ_C	δ_H (*J* in Hz)
1	176.9		176.7	
2	47.9	2.42, m	48.0	2.44, m
3	76.1	4.08, dd (8.0, 8.5)	75.9	4.08, m
4	132.6	5.42, m	132.3	5.42, dd (15.1, 7.2)
5	136.4	5.69, dd (15.5, 8.2)	136.4	5.70, dd (15.5, 8.2)
6	43.4	2.30, td (7.6, 3.5)	43.3	2.31, td (7.4, 3.8)
7	75.8	3.76, m	75.8	3.75, m
8	39.5	1.71, m; 1.51, m	39.2	1.71, m; 1.53, m
9	75.0	3.76, m	75.1	3.75, m
10	44.6	1.51, m	44.2	1.53, m
11	72.4	3.86, m	72.2	3.89, m
12	33.3	1.58, m; 1.36, m	33.5	1.58, m; 1.37, m
13	30.1	1.43, m; 1.31, m	30.3	1.39, m; 1.29, m
14	40.3	1.58, m	40.5	1.58, m
15	72.1	3.86, m	72.3	3.86, m
16	42.0	1.88, m; 1.76, m	41.7	1.80, m;1.77, m
17	99.9		99.8	
18	77.7	3.33, d (9.5)	77.3	3.34, d (9.5)
19	69.7	3.86, m	69.7	3.86, m
20	41.3	1.88, m; 1.31, m	41.2	1.89, m; 1.29, m
21	66.3	4.16, m	65.6	4.08, m
22	44.4	1.71, m; 1.58, m	41.8	1.77, m; 1.71, m
23	65.9	3.86, m	71.0	5.19, m
24	43.7	1.81, m; 1.71, m	44.5	1.71, m
25	71.1	5.22, m	65.8	3.86, m
26	40.4	1.71, m; 1.51, m	43.2	1.58, m; 1.37, m
27	68.9	3.94, m	69.4	4.11, m
28	45.4	1.51, m	45.2	1.53, m
29	75.2	4.04, m	75.7	4.05, m
30	134.9	5.63, dd (15.2, 6.3)	135.2	5.65, dd (15.2, 6.3)
31	132.1	6.16, dd (15.1, 10.3)	131.9	6.18, dd (15.2, 10.3)
32	131.2	6.06, dd (15.1, 10.3)	131.8	6.06, dd (15.1, 10.3)
33	136.9	5.53, dd (15.0, 8.8)	136.8	5.51, dd (15.0, 8.8)
34	40.5	2.54, q (7.6)	40.6	2.53, q (7.7)
35	80.2	4.74, dd (7.6, 4.2)	79.7	4.75, dd (8.1, 3.7)
36	32.7	1.88, m	32.5	1.89, m
37	42.3	1.36, m; 0.93, m	42.3	1.33, m; 0.92, m
38	30.7	1.58, m	30.5	1.58, m
39	37.2	1.31, m; 1.07, m	37.2	1.29, m; 1.06, m
40	27.6	1.36, m	27.7	1.33, m
41	33.8	1.97, quint (6.8)	33.8	1.97, m

42	133.0	5.48, dt (15.0, 7.0)	133.0	5.48, dt (15.0, 7.0)
43	130.0	5.42, m	129.8	5.43, dt (15.0, 7.0)
44	30.7	2.07, q (7.1)	30.6	2.07, q (7.1)
45	29.9	1.64, m	29.9	1.64, m
46	42.0	3.15, t (7.1)	42.0	3.15, t (7.3)
47	15.2	1.01, d (6.9)	14.9	1.01, d (6.9)
48	17.2	1.08, d (6.9)	16.8	1.07, d (6.9)
49	10.6	0.87, d (6.9)	10.6	0.89, d (6.9)
50	15.4	0.91, d (6.9)	15.1	0.91, d (6.9)
51	11.1	0.84, d (6.8)	11.3	0.86, d (6.9)
52	18.0	1.01, d (6.9)	17.8	1.01, d (6.9)
53	14.9	0.91, d (6.9)	14.9	0.91, d (6.9)
54	20.6	0.87, d (6.9)	20.4	0.87, d (6.9)
55	158.2		158.2	
56	28.3	2.83, s	28.3	2.83, s
1’	171.5		171.5	
2’	46.4	3.30, overlap	45.4	3.30, overlap
3’	174.1		174.3	

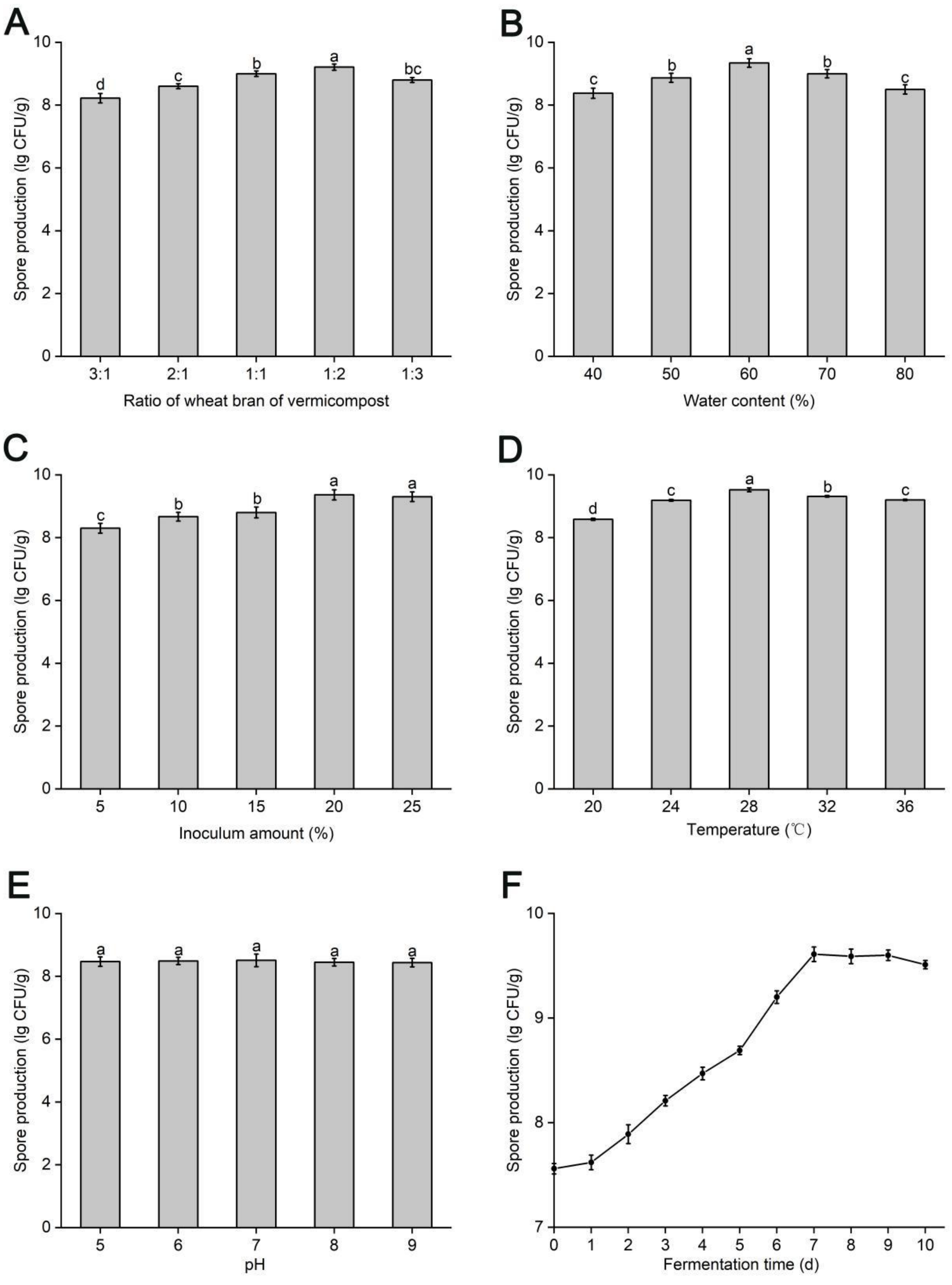

Fig. S1. Effect of vermicompost/wheat bran, water content, inoculum amount, temperature, initial pH and fermentation time on the spore production of NEAU-ZSY13 during fermentation. Data with different lowercase letters are significantly different at the 0.05 probability level.

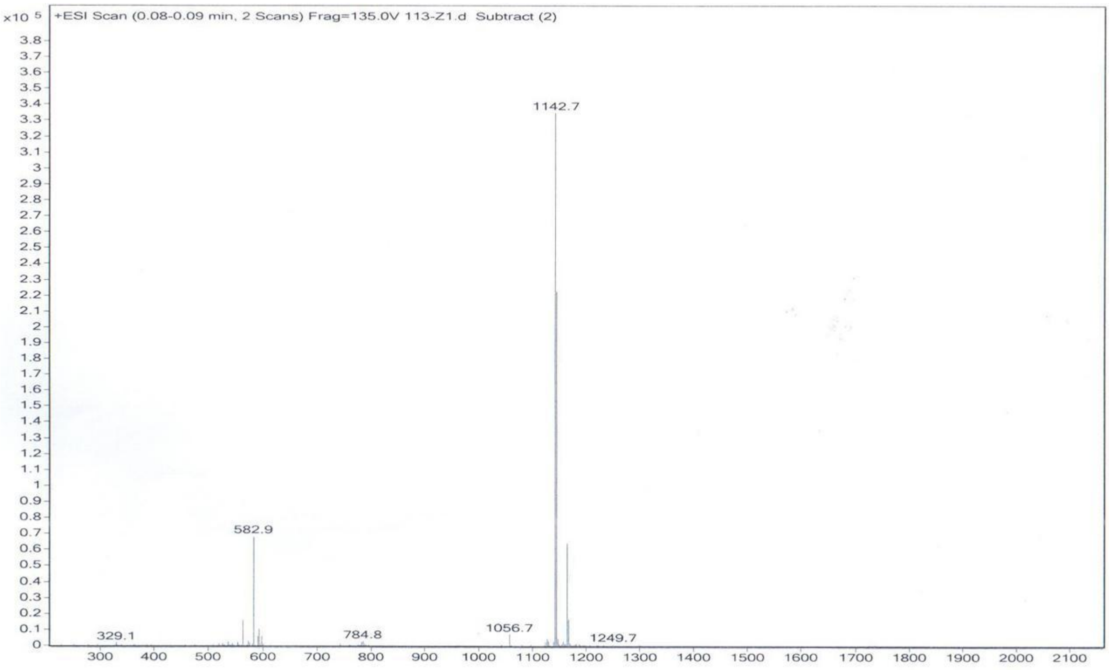

Fig. S2. ESIMS spectrum of compound **1**.

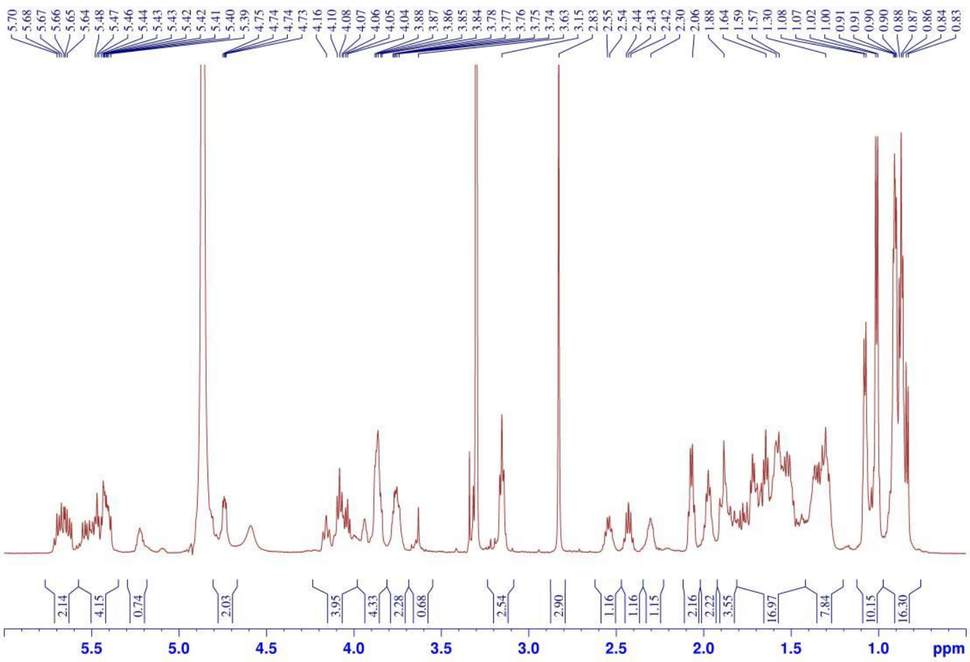

Fig. S3. ^{1}H NMR spectrum of compound **1** in CD_3OD.

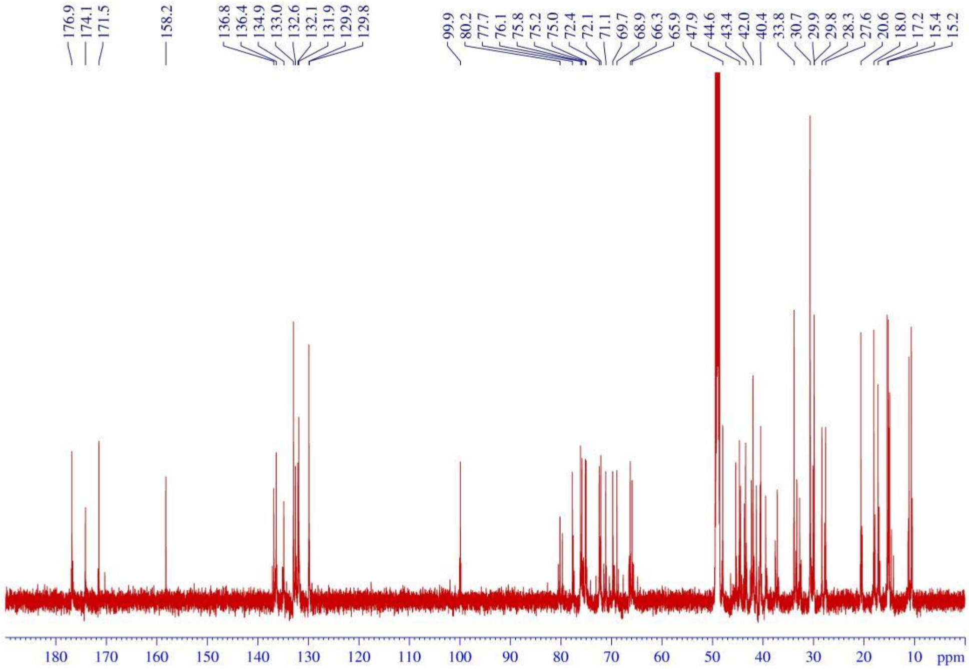

Fig. S4. ^{13}C NMR spectrum of compound **1** in CD_3OD.

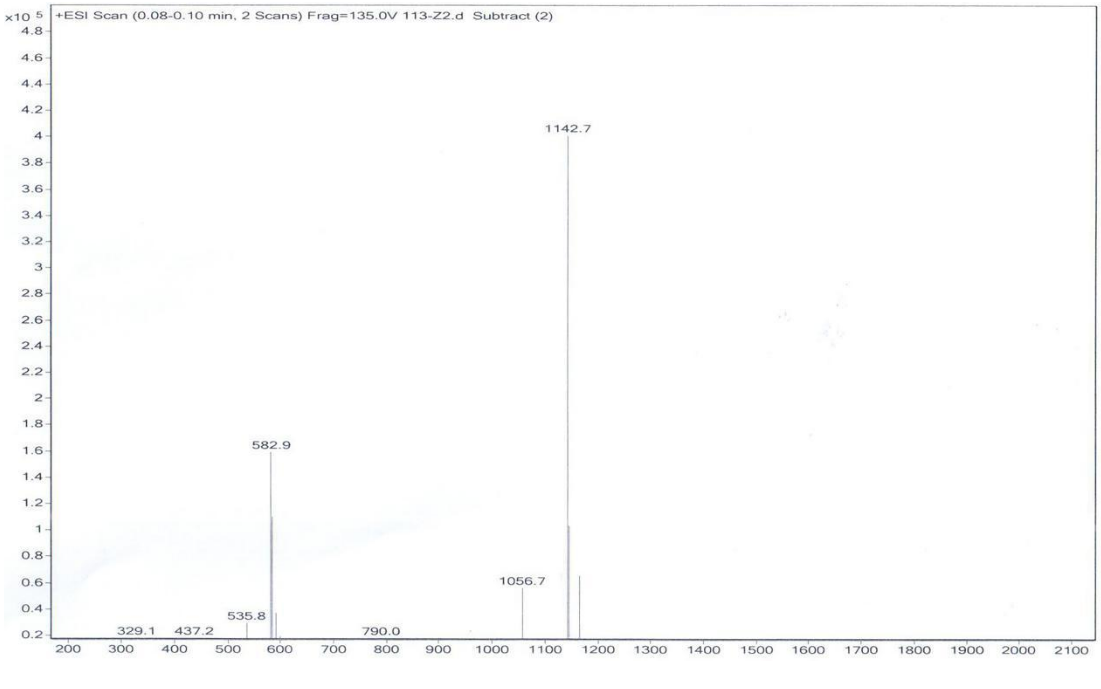

Fig. S5. ESIMS spectrum of compound **2**.

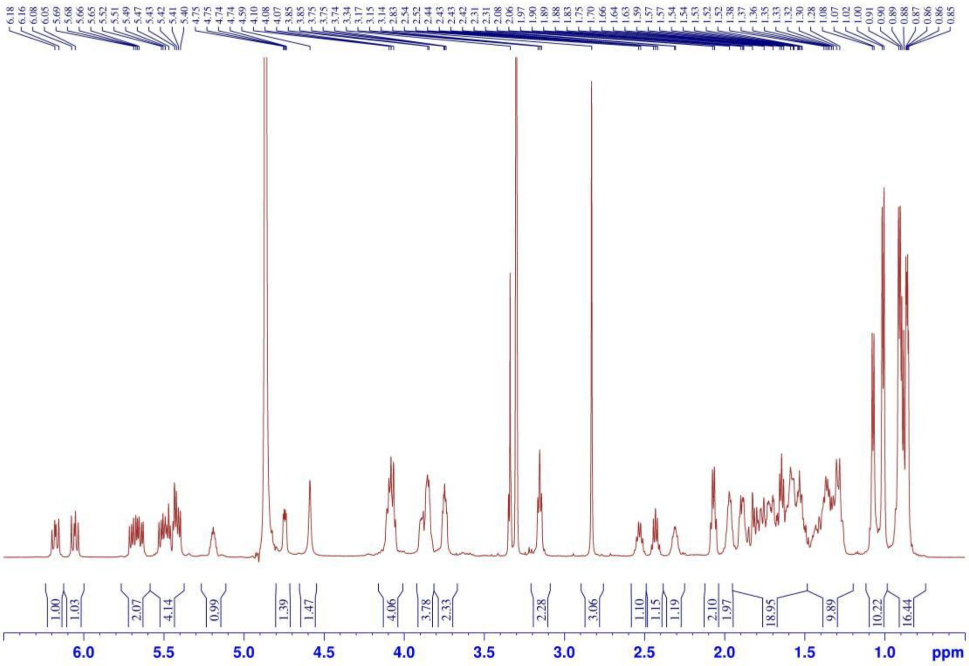

Fig. S6. ^{1}H NMR spectrum of compound **1** in CD_3OD.

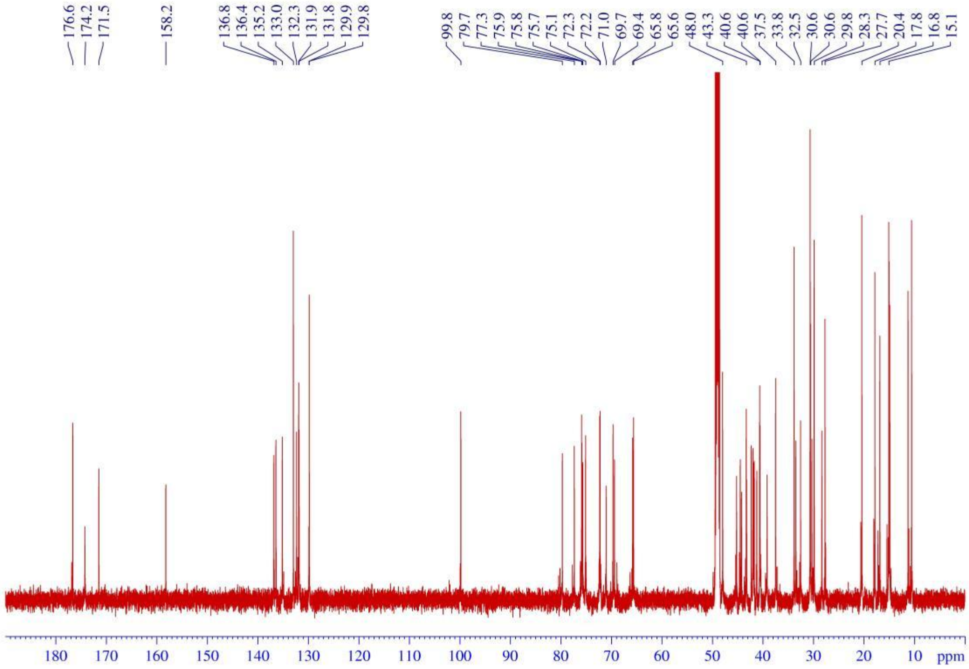

Fig. S7. ^{13}C NMR spectrum of compound **1** in CD_3OD.
